# TSH Receptor Homodimerization in Regulation of cAMP Production in Human Thyrocytes *in vitro*

**DOI:** 10.3389/fendo.2020.00276

**Published:** 2020-04-30

**Authors:** Alisa Boutin, Christine C. Krieger, Bernice Marcus-Samuels, Joanna Klubo-Gwiezdzinska, Susanne Neumann, Marvin C. Gershengorn

**Affiliations:** ^1^Laboratory of Endocrinology and Receptor Biology, National Institute of Diabetes and Digestive and Kidney Diseases, National Institutes of Health Bethesda, MD, United States; ^2^Metabolic Disease Branch, National Institutes of Diabetes and Digestive and Kidney Diseases, National Institutes of Health, Bethesda, MD, United States

**Keywords:** inverted U-shaped dose response curve, cAMP production, phosphoinositide signaling, thyrotropin receptor, TSHR, receptor homodimerization

## Abstract

Thyrotropin hormone (TSH) was reported to exhibit biphasic regulation of cAMP production in human thyroid slices; specifically, upregulation at low TSH doses transitioning to inhibition at high doses. We observed this phenomenon in HEK293 cells overexpressing TSH receptors (TSHRs) but in only 25% of human thyrocytes (hThyros) *in vitro*. Because TSHR expression in hThyros *in vitro* was low, we tested the hypothesis that high, *in situ* levels of TSHRs were needed for biphasic cAMP regulation. We increased expression of TSHRs by infecting hThyros with adenoviruses expressing human TSHR (AdhTSHR), measured TSH-stimulated cAMP production and TSHR homodimerization. TSHR mRNA levels in hThyros *in vitro* were 100-fold lower than in human thyroid tissue. AdhTSHR infection increased TSHR mRNA expression to levels found in thyroid tissue and flow cytometry showed that cell-surface TSHRs increased more than 15-fold. Most uninfected hThyro preparations exhibited monotonic cAMP production. In contrast, most hThyro preparations infected with AdhTSHR expressing TSHR at *in vivo* levels exhibited biphasic TSH dose responses. Treatment of AdhTSHR-infected hThyros with pertussis toxin resulted in monotonic dose response curves demonstrating that lower levels of cAMP production at high TSH doses were mediated by G_i_/G_o_ proteins. Proximity ligation assays confirmed that AdhTSHR infection markedly increased the number of TSHR homodimers. We conclude that *in situ* levels of TSHRs as homodimers are needed for hThyros to exhibit biphasic TSH regulation of cAMP production.

## Introduction

The TSH receptor (TSHR) in human thyroid cells couples to G proteins of all four subfamilies (1), including the stimulatory G protein G_s_, which activates adenylyl cyclase to produce cAMP, G_i_, which inhibits cAMP production, G_13_, which activates p44/42 mitogen-activated protein kinase (MAPK) (2), and G_q_/G_11_, which activate phospholipase C to produce inositol-1,4,5-trisphosphate, which is rapidly degraded to inositol monophosphate (IP-1; phosphoinositide signaling) (3).

TSHR oligomerization in primary cultures of thyrocytes and in cells overexpressing TSHRs has been demonstrated by several techniques including fluorescence resonance energy transfer (FRET) and bioluminescence resonance energy transfer (BRET) (4). Latif et al. demonstrated the existence of TSHR oligomers in intact cells using FRET (5). BRET experiments by Urizar et al. confirmed TSHR homodimerization and demonstrated that the serpentine domain of the TSHR is primarily involved in dimerization (6). Furthermore, this study showed that TSH binding induces negative cooperativity, a mechanism where TSH binding to one site reduces the binding affinity to another site on the TSHR homodimer (6). The potency for TSH to stimulate cAMP signaling is ~100-fold higher than for phosphoinositide signaling (3). We showed previously that the different potencies occurred because cAMP signaling is initiated by binding of one TSH to one protomer of a putative TSHR homodimer whereas phosphoinositide signaling requires binding of two TSH molecules to the TSHR homodimer (7).

We recently reported biphasic regulation of cAMP production by TSH in HEK293 cells overexpressing TSH receptors (HEK-TSHR cells) with upregulation at low doses and downregulation at high doses; that is, an inverted U-shaped dose-response curve (IUDRC) (8). The upregulation was consistent with G_s_-mediated high potency cAMP signaling. The inhibition at high doses of TSH was shown to be mediated by G_i/_G_o_ proteins.

Dumont and co-workers had observed a biphasic response for TSH-mediated cAMP generation in thyroid tissue slices from 11 of 14 patients (9, 10). We also found biphasic cAMP production in hThyros but it occurred in only 25% of the tested hThyros *in vitro*. We had observed previously that the level of TSHRs rapidly decreased after hThyros were placed in cell culture suggesting a potential dependence of the biphasic response on the level of TSHR expression. Therefore, we tested the hypothesis that *in vivo* levels of TSHRs were needed for formation of TSHR homodimers allowing for biphasic cAMP regulation.

## Materials and Methods

### Primary Cultures of hThyros

Primary cultures of hThyros were established by isolating cells from normal thyroid tissue samples from patients undergoing surgery for thyroid tumors at the National Institutes of Health Clinical Center as described previously (11). The studies involving human participants were reviewed and approved by the NIDDK Institutional Review Board. Written informed consent was obtained from the participants of the study. Parts of the thyroid tissue specimens were frozen immediately in liquid nitrogen for subsequent mRNA measurement.

We routinely measure thyroglobulin (TG) as a marker of thyrocyte functionality. In general, TG expression remains detectable for up to 10 passages. In addition, we observe TSH-stimulated upregulation of the expression of other thyroid gene markers, such as thyroid peroxidase (TPO), sodium iodide symporter (NIS), and iodothyronine deiodinase 2 (DIO2).

### Infection With AdhTSHR

Adenovirus expressing full-length human TSHR, TSHR-Ad-RGD, was kindly provided by Basil Rapoport and Sandra McLachlan from Cedars-Sinai Medical Center (12). We refer to this virus as AdhTSHR. We used adenoviral-mediated gene transfer to produce hThyros with markedly increased levels of TSHR. For infection, 2.5 ×10^4^ cells were seeded in 48-well plates in growth medium (DMEM supplemented with 10% FBS (Hyclone Laboratories, Inc., Logan, UT, USA) and penicillin/streptomycin, Mediatech Inc, Manassas, VA, USA) and were incubated in a humidified atmosphere of 5% CO_2_ at 37°C. After cells attached (6–24 h), the medium was aspirated and replaced with growth medium containing 0, 1, 10, or 50 MOI/cell, and the cells were incubated at 37°C for 72 to 96 h. TSHR overexpression was confirmed by quantitative RT-PCR and by fluorescence-activated cell sorting (FACS) analysis (13). Homodimer TSHR formation was measured by proximity ligation assay (PLA). For PLA, cells were reseeded into MatTek glass bottom dishes (MatTek Corporation, Ashland, MA, USA) 48 h after infection with AdTSHR and 24 h before the experiment (see below).

### Measurement of TSHR mRNA Expression

Levels of mRNA were measured in total RNA preparations using RNeasy Mini Kits (Qiagen, Hilden, Germany) followed by reverse transcription to synthesize first-strand cDNA using High Capacity cDNA Archive Kit (Applied Biosystems, Foster City, CA, USA). DNase was used to prevent Ad-TSHR genomic DNA contamination. Quantitative RT-PCR was performed using the prepared cDNA and iTaq™ Universal Probe Supermix (Bio-Rad Laboratories, Hercules, CA, USA) and primers and probes for TSHR were obtained from Taqman, Assay-on-Demand (Applied Biosystems). Quantitative RT-PCR results were normalized to GAPDH as described previously (14).

### Measurement of TSHR Cell Surface Protein Expression

Mouse monoclonal anti-TSHR antibody, KSAb1, was kindly provided by Dr. Paul Banga, Kings College London (15). The human monoclonal TSHR antibodies M22 and 2C11 were purchased from Kronus (Star, ID, USA) and Thermo Fisher Scientific, Inc. (Waltham, MA, USA), respectively.

KSAb1, M22, and 2C11 were labeled with Alexa Fluor 647 using the Alexa Fluor 647 Antibody Labeling Kit (Thermo Fisher Scientific, Inc.) according to manufacturer's directions. 72–96 h post-infection, cells were harvested using Accutase (Innovative Cell Technologies, Inc., San Diego, CA, USA), washed twice, resuspended to 1 ×10^6^ cells/ml in ice-cold HBSS with 2% FBS, and maintained at 4°C. Cells were incubated with KSAb1-647, M22-647, or 2C11-647 at a final concentration of 1 μg/ml. Following a 2 h antibody incubation, cells were washed and resuspended in ice-cold HBSS with 2% FBS. Flow cytometry was performed using a BD FACSAria II Cell Sorter (BD Biosciences, Franklin Lakes, NJ, USA), with a 100-μm nozzle and a sheath pressure of 20 psi. Cell gating was done with FACS Diva software. Debris and clustered cells were excluded from gated populations. Labeled cells were gated to exclude auto-fluorescence as defined by fluorescence of unlabeled cells.

### cAMP Assay

cAMP production was measured in hThyros incubated in HBSS/10 mM HEPES, pH 7.4 containing 0.5 mM 3-isobutyl-1-methylxanthine (IBMX) (Sigma-Aldrich) with increasing doses of bovine TSH (bTSH) (Merck Millipore, Darmstadt, Germany) in a humidified 5% CO_2_ incubator at 37°C for 60 min as previously described (8). Following aspiration of the medium, cells were lysed using lysis buffer of the cAMP-Screen chemiluminescent immunoassay system (Thermo Fisher Scientific Inc.). The cAMP content of the cell lysate was determined using the method described in the manufacturer's protocol.

### Inositol-1-phosphate (IP-1) Assay

IP-1 production, an index of phosphoinositide signaling, was measured as described previously under the same conditions as cAMP production except that we used the stimulation buffer provided with the kit containing 50 mM LiCl (3).

### Measurement of TSHR Homodimer Expression

Proximity ligation assay (PLA) was used to quantify TSHR-TSHR interactions, that is, TSHR homodimers (16). KSAb1 was conjugated to either Duolink PLUS or MINUS oligo arms using Duolink® *In Situ* Probemaker kits (Sigma-Aldrich, St. Louis, MO, USA). Cells were fixed with 4% methanol-free formaldehyde solution for 15 min at room temperature, blocked with Duolink blocking solution for 1 h at 37°C and then incubated with 4 μg/ml KSAb1-PLUS and KSAb1-MINUS overnight at 4°C. PLA was performed according to the manufacturer's directions. Cells were counter-stained with SYTO9 and phalloidin (Thermo Fisher Scientific, Inc) and stored at 4°C prior to fluorescent microscopy. Cells were imaged on a Zeiss 510 NLO/Meta system using a Plan-Apochromat 63x/1.40 Oil DIC objective. Phalloidin fluorescence was used to confirm that Duolink signals were on cells. SYTO9 fluorescence was used to count the number of cells. Cells over-expressing thyrotropin-releasing hormone receptors using AdCMVmTRH-R1 adenovirus (17) were used as control and gave no signal (data not shown). Although PLA is commonly quantified by the number dots/cell, the high number of dots in the 10 and 50 MOI conditions resulted in a coalescence of signal, therefore, dots/cell could not be quantified. Instead, positive PLA signal was quantified by the area of pixels in the PLA channel (546 nm) with fluorescence intensity greater than background. Data were averaged over all images to determine mean ± SEM. Duolink signal per cell was quantified with ImageJ (18, 19).

### Statistical Analysis

The potencies (i.e., EC_50_s) were calculated from the dose-response curves using GraphPad Prism Version 7 for Windows (GraphPad Software, La Jolla, CA, USA). Statistical analysis was performed by Student's *t*-test; statistical significance was defined as *P* <0.05.

## Results

### Comparison of TSHR Expression in Thyroid Tissue and in Primary Cultures of hThyros

**Figure 1:**
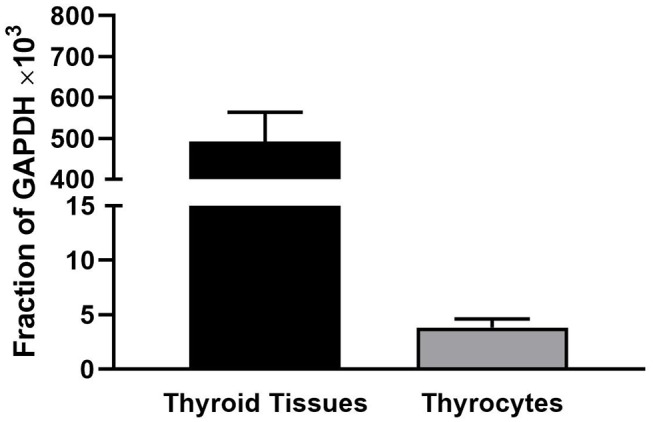
Comparison of the levels of TSHR mRNA in thyroid tissue and hThyros *in vitro*. There were 29 samples of thyroid tissue and 31 samples of hThyros. The mRNA levels were normalized to GAPDH mRNA. The values were markedly different (*P* <0.0001).

We observed that levels of TSHR mRNA in thyroid tissue obtained immediately after thryroidectomy are 100-fold higher than in hThyros *in vitro*. Figure 1 illustrates TSHR mRNA levels in human thyroid tissues (*n* = 29) and in hThyros after at least 2 weeks in culture (*n* = 31), a time at which cells are used in studies of TSH signaling. As a fraction of GAPDH mRNA, thyroid tissue TSHR mRNA was 557 ± 66 ×10^3^ and hThyro TSHR mRNA was 3.8 ± 0.78 ×10^3^ (*P* <0.0001).

**Figure 2:**
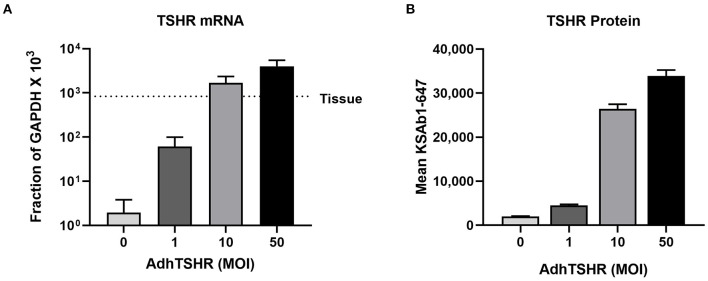
TSHR mRNA and protein expression in hThyros infected with AdhTSHR. **(A)** Effect of increasing AdhTSHR MOI on the level of TSHR mRNA in hThyros in culture. The measurements were performed in thyrocytes from four different donors in duplicate. Data are expressed as mean ± SD. **(B)** hThyros infected with increasing amounts of AdhTSHR were analyzed for KSAb1-647 fluorescence by flow cytometry. Bars depict mean KSAb1-647 fluorescence of labeled cells. Representative data from one thyrocyte strain are shown, and this experiment was repeated in thyrocytes from four different donors. Increase of TSHR protein expression following infection with 50 MOI was donor dependent and ranged from 5 to 63-fold over control (0 MOI).

Figure 2A shows TSHR mRNA levels in hThyros infected with 0, 1, 10, and 50 MOI of AdhTSHR. As expected, there was a direct correlation of TSHR mRNA levels with increasing MOI. At AdhTSHR MOI of 10, the levels of TSHR mRNA in hThyros (1,700 ± 650 as a fraction of GAPDH x 10^3^) were 3.0-fold higher than those in thyroid tissue. Measurement of cell surface TSHR protein expression in hThyros in culture from four different donors by flow cytometry with KSAb1-647 confirmed the correlation between increasing MOI and TSHR expression (Figure 2B). Similar increases in TSHR expression were found with two additional anti-TSHR antibodies M22-647 and 2C11-647 (data not shown). The increase of TSHR protein expression following infection with 50 MOI of AdhTSHR was donor dependent and ranged from 5 to 63-fold over control (0 MOI).

### *in vivo* TSHR Expression Is Crucial for Biphasic cAMP Regulation in hThyros

**Figure 3:**
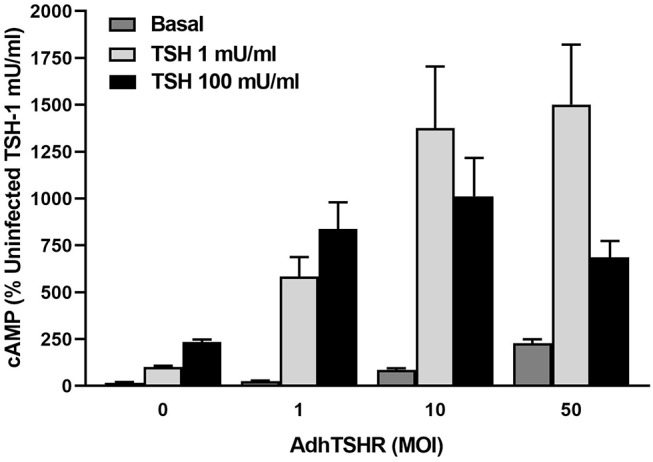
*In vivo* level expression of TSHR is required for biphasic cAMP production induced by TSH in hThyros. Cells were uninfected (0 MOI) or infected with AdhTSHR at 1, 10, and 50 MOI. Cells were not exposed to TSH (Basal) or were stimulated with TSH at 1 or 100 mU/ml. cAMP production was measured in cells incubated in buffer containing IBMX to inhibit cAMP degradation. Results shown are from 2 experiments performed in triplicate and are expressed as mean ± SEM. We observed statistically significant increase in constitutive levels of cAMP with increasing MOI of AdhTSHR (*P* <0.01 between 0 and 1 MOI and *P* <0.0001 between 0 and 10 and 50 MOI). The effects of 100 mU/ml TSH were reduced compared to those of 1 mU/ml at 10 and 50 MOI (*P* <0.045 between 1 and 100 mU/ml with 10 MOI and *P* <0.01 between 1 and 100 mU/ml with 50 MOI) with the decrease in cAMP greater at AdhTSHR of 50 MOI than at 10 MOI (*P* <0.04).

**Figure 4:**
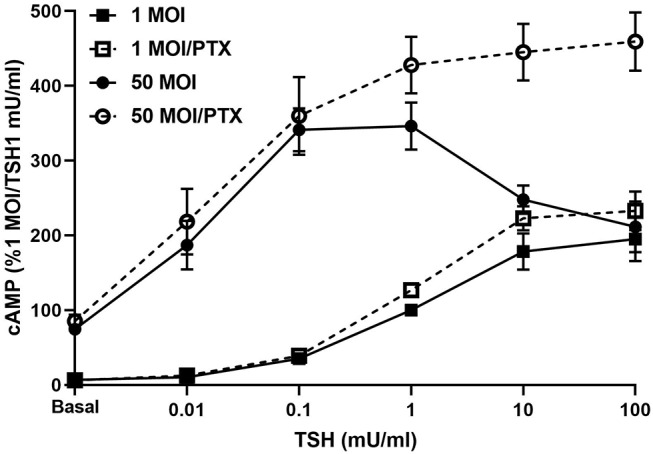
Biphasic cAMP production is stimulated by low TSH doses and inhibited by G_i_/G_o_ at high TSH doses. hThyros were infected with AdhTSHR at 1 MOI (squares) or 50 MOI (circles). Pertussis toxin (100 ng/ml) was added to cells 18 h prior to adding increasing doses of TSH. cAMP production was measured in cells incubated in buffer containing IBMX to inhibit cAMP degradation. Results shown are from 3 experiments performed in duplicate and presented as mean±SEM. In hThyros infected with AdhTSHR at 50 MOI and stimulated with high TSH doses, cAMP production exhibits a classic IUDRC. The decreasing response at 10 and 100 mU/ml TSH is abolished in cells pretreated with pertussis toxin (PTX) (*P* <0.01 and *P* <0.005, respectively).

Infection of hThyros with increasing AdhTSHR MOI caused a progressive increase in basal cAMP levels that is due to constitutive signaling activity of TSHR (20), and concordantly increased cAMP production by 1 mU/ml TSH (Figure 3). In contrast, the effects of 100 mU/ml TSH were blunted compared to those of 1 mU/ml at 10 and 50 MOI with the decrease in cAMP greater at AdhTSHR of 50 MOI than at 10 MOI. These data indicate that the TSH-induced IUDCR for cAMP production may be dependent on the TSHR expression level. Therefore, we followed up by generating full dose response curves with TSH for cAMP production (Figure 4). In hThyros infected with AdhTSHR at 1 MOI, increasing doses of TSH stimulated a monotonic cAMP response that, as expected, was increased at high doses of TSH when the cells were pretreated with pertussis toxin, which relieves inhibition of adenylyl cyclase by G_i_/G_o_ proteins. In contrast, in hThyros infected with AdhTSHR at 50 MOI, increasing doses of TSH stimulated a biphasic cAMP response. As expected, because of the intrinsic constitutive activity of TSHR (20), the basal level and the response to low doses of TSH was greater and with higher potency than that in cells infected with 1 MOI AdhTSHR. More importantly, at high doses of TSH, cAMP production was inhibited exhibiting a classic IUDRC (21, 22). The decreasing response exhibited at high doses of TSH was abolished in cells pretreated with pertussis toxin. Thus, the biphasic response was dependent on high levels of TSHR expression, activation of G_s_ to stimulate adenylyl cyclase and activation of G_i_/G_o_ proteins to dampen adenylyl cyclase activity.

Of note, the responses in IP-1 production to various doses of TSH was monotonic in hThyros infected with 1 or 50 MOI AdhTSHR (Supplemental Figure 1); there was little stimulation of IP-1 production in uninfected cells (data not shown) and low levels of IP-1 production in cells infected with 1 MOI AdhTSHR. As reported before, the potency for TSH in hThyros to stimulate IP-1 signaling is ~100-fold lower than for cAMP signaling. As expected, because of the increased TSHR levels and TSHR homodimer formation, the IP-1 response was greater in 50 MOI AdhTSHR infected cells. There was no effect of pertussis toxin pretreatment as the IP-1 response is mediated primarily by G_q_/G_11_.

### TSHR Levels Found in Thyroid Tissue Are Required for Homodimerization

**Figure 5:**
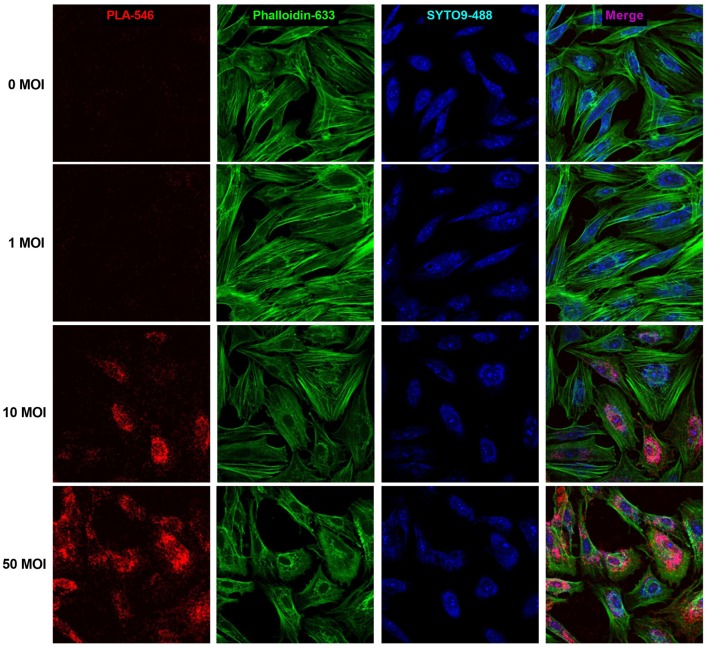
Formation of TSHR homodimers in hThyros infected with AdhTSHR. Increasing protein expression induces the formation of TSHR homodimers. Positive dots (red) in the proximity ligation assay decorate TSHR homodimers. Micrographs represent data from one of three thyrocyte strains from different donors. Cell boundaries were marked using Phalloidin Alexa 633 (green). Cell number was determined by SYTO9 staining (blue). We found no statistically significant difference between 0 and 1 MOI. The differences between uninfected cells and cells infected with 10 and 50 MOI were highly statistically significant (*P* <0.0001).

PLA analysis was carried out to measure formation of TSHR homodimers. In uninfected hThyros, a few positive dots were detected which indicates homodimerization of TSHR at the endogenous expression level (Figure 5). hThyros infected with 1 MOI did not show a significant increase of TSHR homodimer expression compared to uninfected cells. In contrast, the Duolink signals in cells infected with 10 and 50 MOI AdhTSHR were very robust and demonstrated a strong increase in TSHR homodimer formation with correlates with an increase in TSHR expression (Figure 2). Figure 5 is representative of one of three donors. In this PLA, we used KSAb1, which is a monoclonal antibody that binds to a single epitope on TSHR, labeled with positive and negative adducts. Thus, the signal is from TSHR homodimers. We use the term homodimer to represent TSHR-TSHR dimers but recognize there may be higher order oligomers also. Moreover, although the PLA data demonstrate that two TSHR protomers are within 40 nanometers of each other, it does not prove that they are in direct contact.

## Discussion

Much of our understanding of TSHR signaling originated from *in vitro* studies. In those studies, TSH stimulation of cAMP production was monotonic. However, as mentioned above, Laurent et al. had observed a biphasic response for TSH-mediated cAMP generation in thyroid tissue slices from 11 of 14 patients (10). It is likely that TSHR levels in tissue slices were more similar to those present *in vivo* than those found in dispersed monolayer cultures *in vitro* that were employed in previous studies. Our recent report that TSH regulates cAMP in a biphasic manner in HEK 293 cells stably overexpressing the TSHR (HEK-TSHR cells) (8) implied that past investigations in monolayer cells may have missed an aspect of TSHR signaling that may be prevalent *in vivo*. We showed that TSHR expression is much higher in tissue obtained at thyroidectomy than in hThyros *in vitro*. We think that lower TSHR expression *in vitro* resulted in a blunted signaling response in primary cultures of hThyros rather than a different signaling profile. This possibility underscored the importance of formally testing the hypothesis that *in vivo* levels of TSHRs were needed for biphasic cAMP regulation. Previously, we reported that biphasic regulation of cAMP by TSH occurred robustly in HEK-TSHR cells (8) but we could only observe it in a small fraction of hThyros strains. Since the IUDRC was observed in hThyros from some donors, the findings in the HEK-TSHR cells could not be dismissed and motivated us to determine whether the IUDRC was an artifact of HEK-TSHR cells or a biologically relevant occurrence as it had also been described earlier by Dumont and co-workers for thyroid slices (9, 10). Here we found that in the majority of uninfected hThyros, cAMP production was monotonic with a plateau at TSH levels above 10 mU/ml. In contrast, hThyro strains from four different donors infected with AdhTSHR expressing TSHR mRNA at near *in vivo* levels exhibited higher levels of cAMP production that were biphasic with maximal levels with 1 mU/ml TSH and decreased production at higher TSH doses. We do not have a good measure of cell surface TSHR protein *in vivo* and, therefore, cannot conclude that we have replicated functional TSHRs *in vitro* at a level similar to that found *in vivo*. Nevertheless, we think we can conclude that near *in vivo* levels of TSHRs are needed for hThyros to exhibit biphasic TSH regulation of cAMP production.

Organization as dimers (or higher order oligomers) is a common feature of GPCRs that has been demonstrated for TSHR (4–6). It is well-documented that dimerization can have a major influence on signaling properties of interacting protomers in ligand binding, G protein coupling selectivity, and signal transduction mechanisms or cell surface expression (23). Here we show that TSHR dimers are likely involved in the mechanism of reduced cAMP production at high TSH doses. Because KSAb1 is a monoclonal antibody with a single epitope on TSHR that would not allow two KSAb1 antibodies to bind to a single receptor protomer, the PLA using KSAb1 with plus or minus arms allows us to positively identify TSHR homodimers. The PLA data presented herein (Figure 5) demonstrate that a high number of TSHR homodimers is present in hThyros expressing *in vivo* levels of TSHR. The decreasing phase of the biphasic dose response curve of TSH-stimulated cAMP production occurs at TSH doses similar to those needed for activation of G_q_/G_11_. As we showed previously (7), signaling via cAMP production by activation of G_s_ requires occupancy of TSHR homodimers by one TSH molecule to one of the two protomers whereas activation of G_q_/G_11_ leading to phosphoinositide signaling requires occupancy by two TSH molecules thereby explaining the ~100-fold lower TSH potency for phosphoinositide vs. cAMP signaling. Negative cooperativity was previously shown to be dependent on TSHR homodimerization (6), that is, on occupancy of the binding site on the second protomer of the homodimer. The dose dependency of TSH stimulation of inositol monophosphate (IP1) production was found similar to that of negatively cooperative ^125^I-TSH binding, which is known to require binding of unlabeled TSH to the low-affinity site on the second protomer (7). The need for the higher doses of TSH to bind to the unoccupied protomer is caused by the negative cooperativity since the occupied protomer induces the lower affinity exhibited by the second protomer. In this study, we show that the decreasing phase of cAMP production is reversed by pertussis toxin blocking of G_i_/G_o_ activation in hThyros as we demonstrated previously in HEK-TSHR cells (8). Since both high TSH doses and TSHR homodimers are necessary for cAMP inhibition, we conclude that G_i_/G_o_ activation by TSH requires binding of two TSH molecules, one to each TSHR protomer of TSHR homodimers as for G_q_/G_11_ activation.

Hormesis, a biphasic response to increasing doses of a substance or condition, has been found in biology in general, especially in toxicology. The occurrence of U-shaped dose-response relationships has been documented in numerous biological, toxicological, and pharmacological investigations. Many of the endpoints studied are of considerable significance to public health (e.g., body weight, cholesterol levels, ethanol consumption, longevity, cancer incidence, cognitive functions and memory). An inverted IUDRC is a typical example of hormesis, and several hormetic dose-response relationships have been reported in receptor-mediated cell signaling (21). These findings suggest that hormetic responses may mediate specific cellular or physiological pathways. For stimulation of TSHRs, we suggest that the biphasic TSH dose response may be a regulatory mechanism to limit overstimulation of thyroid gland function. The mechanism we describe herein can serve as yet another fine regulator, in addition to the hypothalamic-pituitary-thyroid axis classical negative feedback, which emphasizes the importance of maintaining homeostasis in this axis controlling development, energy metabolism, growth, reproduction as well as heart and digestive function, muscle control, brain development, mood and bone maintenance.

In conclusion, we have shown that generation of a biphasic cAMP dose response by TSH in hThyros is dependent on the level of TSHR expression and the formation of TSHR homodimers. Given the likely higher TSHR expression in thyrocytes in the thyroid gland, signaling involving TSHR homodimers may have more biological relevance than previously appreciated.

## Data Availability Statement

The datasets generated for this study are available on request to the corresponding author.

## Ethics Statement

The studies involving human participants were reviewed and approved by the NIDDK Institutional Review Board. Written informed consent was obtained from the participants of the study.

## Author Contributions

AB and CK: designed research, performed experiments, analyzed and interpreted the data, wrote the manuscript. BM-S: performed experiments, analyzed and interpreted the data. JK-G: provided thyroid tissue samples and facilitated collaboration with clinicians. SN: designed research, interpreted the data, wrote the manuscript. MG: designed and supervised the research, interpreted the data, wrote the manuscript.

## Conflict of Interest

The authors declare that the research was conducted in the absence of any commercial or financial relationships that could be construed as a potential conflict of interest.

## Supplementary Material

Supplemental Figure 1 Effects of hThyros infection with 1 and 50 MOI AdhTSHR and pretreatment with pertussis toxin on TSH-stimulated IP-1 production. Cells were infected with AdhTSHR at 1 MOI (squares) or 50 MOI (circles). Pertussis toxin (100 ng/ml) was added 18 h prior to adding TSH. IP-1 production was measured in cells incubated in buffer containing LiCl to inhibit IP-1 degradation. Results shown are from 3 experiments performed in duplicate and presented as mean SEM. The IP-1 response was greater in 50 MOI AdhTSHR infected cells (P <0.006, P <0.002, and P <0.003 for 1, 10, and 100 mU/ml TSH). There was no effect of pertussis toxin (PTX) pretreatment.
