# Supplementary figures and images for: TSH Receptor Homodimerization in Regulation of cAMP Production in Human Thyrocytes in vitro
Source: Front Endocrinol (Lausanne). 2020 Apr 30;11:276. doi: 10.3389/fendo.2020.00276 (PMC7203478; doi:10.3389/fendo.2020.00276)

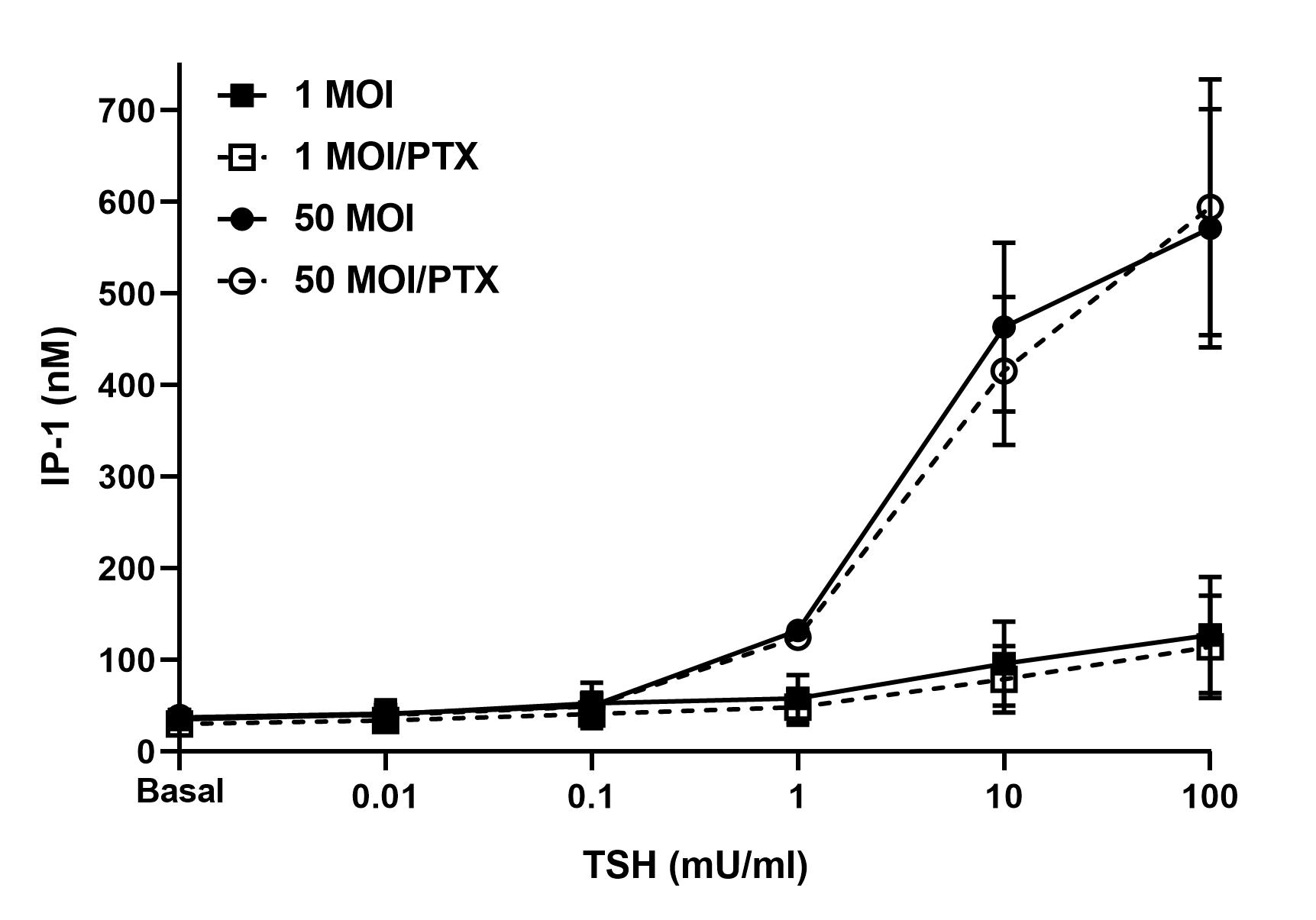

Supplement: Supplemental Figure 1 — Effects of hThyros infection with 1 and 50 MOI AdhTSHR and pretreatment with pertussis toxin on TSH-stimulated IP-1 production. Cells were infected with AdhTSHR at 1 MOI (squares) or 50 MOI (circles). Pertussis toxin (100 ng/ml) was added 18 h prior to adding TSH. IP-1 production was measured in cells incubated in buffer containing LiCl to inhibit IP-1 degradation. Results shown are from 3 experiments performed in duplicate and presented as mean SEM. The IP-1 response was greater in 50 MOI AdhTSHR infected cells (P <0.006, P <0.002, and P <0.003 for 1, 10, and 100 mU/ml TSH). There was no effect of pertussis toxin (PTX) pretreatment. [file Image_1.jpeg]
